# Clinicopathological Features of Inflammatory Myofibroblastic Tumor in the Breast

**DOI:** 10.1155/2022/1863123

**Published:** 2022-08-12

**Authors:** Shifei Liu, Rui Yuan, Yulan Jin, Chunyan He, Xingzheng Zheng, Yang Zhan

**Affiliations:** ^1^Department of Pathology, Beijing Obstetrics and Gynecology Hospital, Capital Medical University, Beijing Maternal and Child Health Care Hospital, 17 Qihelou Street, Dongcheng District, Beijing 100006, China; ^2^Medical School of Chinese PLA, 28 Fuxing Road, Beijing 100853, China; ^3^Department of Critical Care Medicine, Chinese PLA General Hospital, Beijing 100853, China

## Abstract

Inflammatory myofibroblastic tumor (IMT) is a mesenchymal spindle cell tumour with low malignant potential which is extremely rare in breasts. Because of the lack of typical imaging and clinical characteristics of IMT, it is easy to misdiagnose before operation. We now report a case of a 37-year-old woman presenting with a mass in her left breast. Ultrasound showed a well-circumscribed lesion in the lower outer quadrant. The patient underwent lumpectomy, and histopathology revealed a tumor which was composed of fusiform cells and inflammatory cells. Immunohistochemistry (IHC) showed tumor cells are positive for vimentin, ALK, BCL2, and SMA. The FISH test demonstrated ALK (2p23) chromosomal translocation (ALK positive). The final diagnosis of breast IMT was rendered with nonclassical morphology. Postoperative 30-month follow-up no evidence showed residual tumor or recurrence. As a very rare tumor, breast IMT could be easily misdiagnosed clinically and pathologically. Complete surgical resection of the tumor is preferred, and it has the risk of recurrence and metastasis.

## 1. Introduction

Inflammatory myofibroblastic tumor (IMT) is a rare myofibroblastic neoplasm that most frequently occurs in children and young adults. The tumor is composed of spindle-shaped myofibroblasts with fascicles or storiform patterns and sometimes within loose myxoid stroma. The inflammatory cell infiltrate is common, including plasma cells, lymphocytes, as well as variable numbers of eosinophils and neutrophils [1]. The synonyms of IMT include inflammatory pseudotumor, xanthogranuloma, plasma cell granuloma, and plasma cell pseudotumor [2]. Although IMT more commonly occurs in the lung, mesentery, omentum, and retroperitoneum, but it may also be observed in the liver, spleen, thyroid, gastrointestinal tract, genitourinary tract, and central nervous system [1, 2]. IMT in the breast is rare. Due to the lack of typical imaging and clinical features of breast IMT, it can mimic the other breast neoplasm such as carcinoma or fibroadenoma [3]. Here, we report a new case of a 37-year-old female patient with breast IMT, presenting all the clinical, morphological, immunohistochemical, and molecular pathological features of this rare tumor and briefly discuss all the rare tumors published in PubMed. To our knowledge, this is the most comprehensive discussion of breast IMT reported so far.

## 2. Case Presentation

**Figure 1:**
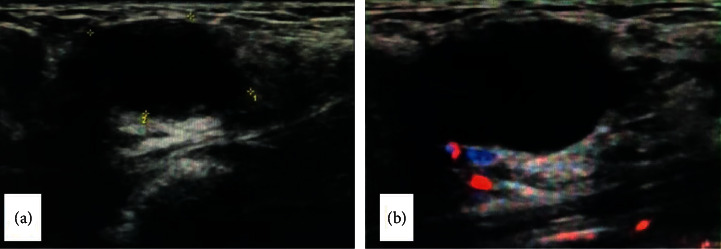
Ultrasonic examination. (a): hypoechoic nodule of the breast with a clear boundary; (b): no obvious blood flow signal in CDFI.

A 37-year-old female patient presented with incidental finding of a mass in her left breast. Her grandfather died of leukemia, and her aunt discovered breast cancer five years ago. Ultrasound showed a 2.1 cm × 1.3 cm mass in the lower outer quadrant of the left breast, with hypoechoic, clear boundary, and no obvious blood flow signal by color doppler flow imaging (CDFI) (Figure 1), indicating a BI-RADS grade 3 breast solid lesion. The surgeon considered fibroadenoma. Lumpectomy was performed in December 2019 and was submitted for pathology examination.

**Figure 2:**
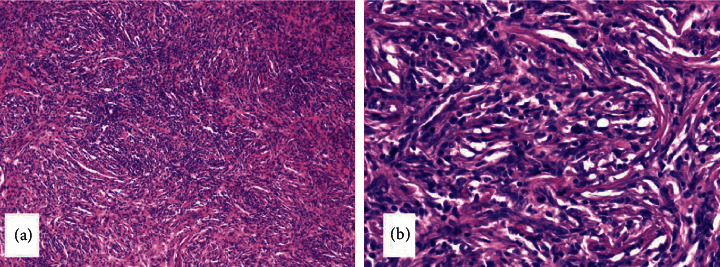
The tumor was composed of spindle cells and inflammatory cells. The spindle cells were arranged in bundles or spirals. HE100× (a); HE400× (b).

**Figure 3:**
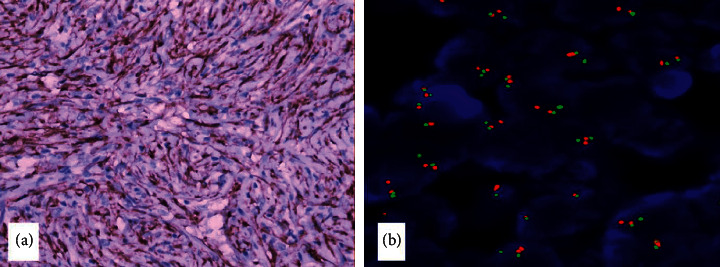
ALK positive expression in spindle tumor cells. IHC 400×. (b): the testing result of ALK by separation probes of FISH, showing ALK gene rearrangement (red and green signals were separated).

Gross examination showed a capsulated, 2.5 cm × 2 cm × 1 cm mass with yellow, tan, and firm cut surface like the fibroadenoma. Microscopically, the mass was composed of spindle cells and formed a storiform pattern, admixing diffuse lymphocytes and the plasma cell infiltrate (Figure 2). The lobular structure of the mammary gland could be seen locally. At the edge, the tumor infiltrated into the fat tissue in some areas. The mitotic figures were about 0-1/10HPF, and no pathological mitotic figures were seen. Although the tumor did not have obvious malignant features, it was necessary to exclude well-differentiated metaplastic carcinoma and make differential diagnosis in a variety of spindle cell tumors of the breast. Finally, immunohistochemical staining showed that the expression of the protein was positive. It is necessary to exclude well-differentiated metaplastic carcinoma and differentiate it from a variety of spindle cell tumors of the breast. Immunohistochemistry (IHC) showed that tumor cells were strong and diffusely positive for ALK (Figure 3(a)), BCL-2, vimentin, P16, and patchy/weakly positive for ER, SMA, P53, and CD34. The Ki67 index was 10%. They were negative for PR, CK, E-cadherin, Her2, EGFR, S-100, desmin, and P63. Fluorescence in situ hybridization (FISH) revealed gene fusion of 2p23 (ALK) (Figure 3(b)). In situ hybridization was EBER negative. Based on these findings, the pathological diagnosis of breast IMT was established. The tumor did not show the most classic and common myxoid pattern of IMT but showed a spindle cell rich type.

Six months past surgery, the patient underwent reresection due to the irregular hypoechoic area around the previous surgery site by ultrasound. However, pathology showed postoperative inflammation and reactive hyperplasia, and there were no tumor recurrence. Till now, the patient has been followed up for 30 months. There is no evidence of recurrence.

## 3. Discussion and Conclusion

IMT is a rare mesenchymal tumor, composed of spindled or stellate myofibroblastic cells and accompanied by inflammatory cell infiltration. The pathogenesis of IMT is unclear. At first, the disease was considered as a nonneoplastic inflammatory disease, which may be related to surgery, trauma, inflammatory infection, and other factors. IMT in the breast is mostly spontaneous, and trauma, surgery, or autoimmune disease-related IMT are occasionally seen [4, 5]. Studies have found that about 50%–75% of IMT have a fusion of ALK with TPM3 and TPM4 genes on 2p23, leading to ALK overexpression, which further supports that IMT is a true tumor rather than a simple inflammatory process [6]. In 2002, the World Health Organization (WHO) officially named it IMT and defined it as an intermediate, occasionally metastatic, and locally recurrent tumor.

In 1988, an IMT occurring in the breast was first reported by Pettinato et al. [7] and was named plasma cell granuloma (inflammatory pseudotumor). Up to now, only 36 cases of breast IMT have been retrieved in PubMed, with onset ages ranging from 13 to 86 years, with the average age of 45.1 years (Table 1). This is the first case of breast IMT found in the pathology department of our hospital in the past ten years.

Breast IMT is more common in women and rarely occurs in men [4, 30]. It mostly occurs in the unilateral breast and can occur in any quadrant of the breast. The site of the current case was in the left lower outer quadrant. Kovacs et al. [28] reported one case of IMT occurring in the nipple of a pregnant patient. Patient's symptoms are atypical, often due to the touch of breast mass, including breast pain, afternoon low fever, night sweat, lymph node enlargement, anemia, and other symptoms. As a low-grade or potentially malignant tumor, breast IMT carries a risk of recurrence and metastasis. Up to now, 6 cases of breast IMT reported had recurrence and metastasis after the first operation, including recurrence of the primary site of tumor, recurrence of the bilateral breast [10, 15], axillary lymph node metastasis [25], inguinal metastasis [2], and supraclavicular lymph node metastasis, and adjacent rib destruction was also reported [27]. Inoue et al. [32] reported a case of IMT of the breast with simultaneous intracranial, lung, and pancreas involvement. In our case, 6 months after operation, ultrasound showed irregular hypoechoic areas in the local area, but only postoperative inflammation and reactive hyperplasia were found in the postoperative pathology, and no residual or recurrence of tumor was found.

Breast IMT imaging often shows nodular or lobulated mass, sometimes with uneven texture or unclear margins. The mass can show rich vasculatures, adhesions, and local compression, so it could be easily misdiagnosed as breast cancer clinically. X-ray examination shows high density shadow, unclear boundary, with or without punctate calcification in the mass; color Doppler ultrasonography shows hypoechoic mass with irregular margins and little or no blood flow signals. The BI-RADS classification is mostly above grade 4A [18, 19, 24, 26, 27]. MRI shows that the mass is irregular, with uneven and rapid enhancement, and mostly demonstrates the morphological and hemodynamic characteristics of breast malignant tumors. Therefore, it is difficult to identify IMT of the breast by image and is easily misdiagnosed as breast malignant tumors preoperatively.

Grossly, the breast IMT tumor is nodular or lobulated, without a capsule or with a pseudoenvelope. The cut surface is often tan/white and firm and may be accompanied by mucinous changes, hemorrhage, and calcification. Microscopically, the tumor is composed of proliferated myofibroblastic cells with a spindle or stellate shape and vesicular nuclei. Usually, they are in fascicles or a storiform growth pattern with inflammatory cells infiltrating the stroma, predominantly plasma cells, lymphocytes, and variable numbers of eosinophils and neutrophils. In addition to spindle cells, round-like histiocyte-like cells can be seen in the tumor. In some cases, irregular, polygonal, or bizarre cells can be seen. Eosinophilic or basophilic inclusions can be seen in the nucleus, similar to ganglion cells or R-S cells. The mitotic figures can vary from region to region. Gobbi et al. [12] reported a case of breast IMT with mixed giant vacuoles and spindle cells and infiltration of inflammatory cells. Highly atypical polygonal cells with large ganglion-like cells are seen in some cases of malignant transformation [6]. Vecchio et al. [4] found that in addition to spindle cells, significant large pleomorphic cells could be seen. In WHO classification, IMT is divided into three basic histological patterns [34]. (1) The myxoid pattern/mucin rich type is the most common; the tumor cells are loosely arranged in myxoid stroma. A large number of blood vessels and inflammatory cell infiltration can be seen, which is similar to nodular fasciitis or granulation tissue in morphology. (2) Hypercellular pattern/spindle cell rich type: compact spindle myofibroblasts with interstitial infiltration of histiocyte-like cells and inflammatory cells, similar to fibrous histiocytoma or leiomyoma; (3) hypocellular fibrous pattern/sclerotic type: the tumor hypocellular with prominent hyalinized stroma may be keloid-like, occasionally calcified, ossified, similar to fibromatosis. According to the above classification, the current case did not show the most classic and common myxoid pattern of IMT but was spindle cell rich type, which brought difficulties in the pathological diagnosis.

Although histological morphology is helpful in identifying IMT, there are no prognostic differences among those subtypes, and sometimes these morphologies can also occur in the same case in practice, and it is not advocated to divide IMT into various subtypes. Epithelioid inflammatory myofibroblastic sarcoma (EIMS) is a rare and aggressive IMT subtype with plump epithelioid or histiocytoid tumour cells with vesicular chromatin, prominent nucleoli, and amphophilic or eosinophilic cytoplasm, often admixed with neutrophils in a rich myxoid stroma [34].

IMT has no specific immunohistochemistry markers. All cases are diffusely strong positive for vimentin, most cases express alpha-SMA, MSA, or desmin, and about 50% of cases express ALK [35]. In addition to the above positive reaction factors, some studies found that S-100, CK, CD21, CD35, and CD34 were negatively expressed in breast IMT, which was similar to IMT expression in other parts [23]. In this case, vimentin, SMA, and ALK were all positively expressed, while CD21, CD23, CD35, and S-100 were negatively expressed, which supported the diagnosis of IMT. Zhou et al. [23] first recorded the detection of ALK protein by IHC and FISH in breast IMT cases and found the overexpression of ALK protein and the gene amplification of ALK-positive breast IMT. Immunohistochemical ALK protein was positive in this case, and the FISH test showed ALK (2p23) chromosomal heterotopia (ALK positive). Since breast IMT has only been reported in individual cases, the diagnostic significance of ALK expression in breast IMT has not been clearly established. At present, it is not clear whether ALK positivity has a certain impact on the prognosis of breast IMT patients, which requires a large number of ALK-positive breast IMT patients to judge its impact on metastasis and recurrence.

Breast IMT should be differentiated from other spindle cell tumors of the breast: (1) myofibroblastoma: benign breast stromal tumor arising from myofibroblasts. Microscopically, uniform spindle cells were arranged in intersecting bundles and separated by hyalinized bands. Varying amounts of adipose tissue are also seen. Tumor cells are positive for desmin, CD34, and hormone receptors. (2) Fibromatosis: composed of long fascicles of spindle cells infiltrating around normal ducts and lobules. Lymphocytic aggregates are usually at the periphery of the tumor. Tumor cells are positive for *β*-catenin in 60–80% of cases and negative for ALK. (3) Nodular fasciitis: mostly located at the superficial part of the upper limb and trunk, with a history of rapid growth. Similar to IMT histomorphology, nodular fasciitis is composed of haphazardly arranged proliferated myofibroblasts, accompanied by myxoid stroma and inflammatory cells. Red cell extravasation is characteristic. The immature fibroblasts are different in size, irregular in shape, and mitotic figures are common. Actin and desmin are usually negative. (4) Spindle cell carcinoma of the breast: belongs to one kind of metaplastic carcinoma of the breast. Tumors are spindle-shaped, loosely arranged, with different shapes, wavy and feathery, accompanied by inflammatory cell infiltration, squamous epithelial metaplasia, and occasional mitotic figures, but the nucleus is often accompanied by moderate-severe polymorphism. Spindle cell carcinomas of the breast have at least one keratin-positive expression, and p63 is expressed in most breast metaplastic carcinomas.

Breast IMT is a rare tumor and could be easily misdiagnosed as breast malignancy due to its atypical clinical symptoms and imaging features, and biopsy is needed for the correct diagnosis. For breast IMT, complete surgical resection of the tumor is preferred. In recent years, more and more reports have shown that alternative therapies such as chemoradiotherapy, targeted therapy, nonsteroidal anti-inflammatory drugs, and steroids have also achieved some results. It has been reported that for relapsed and inoperable IMT, the tumor shrinks significantly after chemotherapy [36]. Despite the unclear etiology and pathogenesis, more and more scholars in recent years tend to believe that the tumorigenesis is caused by alterations in the ALK gene and find that targeted drug therapy is effective in some cases, so ALK may become a potential therapeutic target for breast IMT.

Consequently, as a very rare tumor, breast IMT could be easily misdiagnosed clinically and pathologically. Complete surgical resection of the tumor is preferred, and it has the risk of recurrence and metastasis.

## Figures and Tables

**Table 1:** Clinical features of the breast IMT cases in PubMed.

Case no	Reference no	Year of publication	Age	Gender	Side	Size (mm)	Follow-up (month)	Outcome
1	[7]	1988	29	F^①^	Right	45	30	NED^③^
2	[8]	1995	13	F	Right	40	12	NED
3	[9]	1995	38	F	Right	10	12	NED
4	[10]	1997	66	F	Left	30	14	Bilateral recurrence at 5^th^ month; now NED at 9 months
5	[11]	1997	16	F	Right	20	12	NED
6	[11]	1997	46	F	Right	20	12	NED
7	[11]	1997	18	F	Right	80	6	NED
8	[12]	1999	86	F	Left	15	NA^④^	NA
9	[13]	2002	64	F	Right	30	33	NED
10	[14]	2003	31	F	Right	18	NA	NA
11	[15]	2003	79	F	Right	15	108	Bilateral multiple recurrences in 9 years
12	[16]	2005	60	F	Right	10	85	NED
13	[1]	2005	33	F	Left	20	12	Local recurrence at 3 months
14	[1]	2005	75	F	Left	30	14	NED
15	[1]	2005	47	F	Right	NA	12	NED
16	[5]	2005	46	F	Left	21	12	NED
17	[17]	2007	38	F	Left	10	12	NED
18	[18]	2009	60	F	Left	15	24	NED
19	[19]	2009	47	F	Right	35	36	NED
20	[20]	2010	53	F	Right	35	NA	NA
21	[4]	2011	22	M^②^	Left	70	10	NED
22	[21]	2011	54	F	Left	27	4	NED
23	[2]	2013	56	F	Right	40	10	Local recurrence at 3, 7, and 10 months
24	[22]	2013	39	F	Left	40	24	NED
25	[23]	2013	46	F	Right	11	NA	NA
26	[24]	2014	23	F	Left	20	12	NED
27	[25]	2014	56	F	Right	90	5	Local recurrence at 2 months
28	[26]	2015	67	F	Left	10	6	NED
29	[27]	2015	27	F	Right	30	24	Local recurrence at 12 months
30	[28]	2015	31	F	Left	16	60	NED
31	[29]	2016	38	F	Left	15	16	NED
32	[30]	2017	60	M	Left	15	6	NED
33	[3]	2018	43	F	Left	12	12	NED
34	[31]	2018	52	F	Right	50	8	NED
35	[32]	2018	16	F	Right	22	9	NED
36	[33]	2021	50	F	Right	45	44	NED

^①^F, female; ^②^M, male; ^③^NED, no evidence of disease; ^④^NA, not available.

## Data Availability

All the data generated or analysed during this study are included within this published article.

## Abbreviations

CDFI: Color doppler flow imaging
EIMS: Epithelioid inflammatory myofibroblastic sarcoma
FISH: Fluorescence in situ hybridization
IHC: Immunohistochemistry
IMT: Inflammatory myofibroblastic tumor
WHO: World health organization.
